# Membrane pools of phosphatidylinositol-4-phosphate regulate KCNQ1/KCNE1 membrane expression

**DOI:** 10.1038/s42003-021-02909-1

**Published:** 2021-12-14

**Authors:** Chen Braun, Xiaorong Xu Parks, Haani Qudsi, Coeli M. B. Lopes

**Affiliations:** Aab Cardiovascular Research Institute, Department of Medicine, University of Rochester School of Medicine and Dentistry, 601 Elmwood Avenue, Rochester, NY 14642 USA

**Keywords:** Cardiovascular genetics, Endocytosis

## Abstract

Plasma membrane phosphatidylinositol 4-phosphate (PI4P) is a precursor of PI(4,5)P_2_, an important regulator of a large number of ion channels. Although the role of the phospholipid PI(4,5)P_2_ in stabilizing ion channel function is well established, little is known about the role of phospholipids in channel membrane localization and specifically the role of PI4P in channel function and localization. The phosphatidylinositol 4-kinases (PI4Ks) synthesize PI4P. Our data show that inhibition of PI4K and prolonged decrease of levels of plasma membrane PI4P lead to a decrease in the KCNQ1/KCNE1 channel membrane localization and function. In addition, we show that mutations linked to Long QT syndrome that affect channel interactions with phospholipids lead to a decrease in membrane expression. We show that expression of a LQT1-associated C-terminal deletion mutant abolishes PI4Kinase-mediated decrease in membrane expression and rescues membrane expression for phospholipid-targeting mutations. Our results indicate a novel role for PI4P on ion channel regulation. Our data suggest that decreased membrane PI4P availability to the channel, either due to inhibition of PI4K or as consequence of mutations, dramatically inhibits KCNQ1/KCNE1 channel membrane localization and current. Our results may have implications to regulation of other PI4P binding channels.

## Abstract (web summary)

Braun et al. investigate the effects of PI 4-kinases and their lipid product, PI4P, in KCNQ channel function and sub-cellular localization. These results suggest a possible harmful effect of PI4K inhibition on cardiac electrophysiology and suggest the distal C-terminus of the channel is critical for PI4P regulation of the KCNQ1/KCNE1 channel.

## Introduction

Phosphatidylinositol 4-kinases (PI4Ks) synthesize phosphatidylinositol 4-phosphate (PI4P). Plasma membrane PI4P is a precursor of PI(4,5)P_2_, an important regulator of a large number of ion channels. PI4P is also known to be present in intracellular membrane compartments, such as the membranes of Golgi and trans-Golgi network and regulates trafficking to and from the Golgi^1,2^. In mammals there are four different PI4K enzymes, two type II enzymes (PI4KIIα and PI4KIIβ) and two type III enzymes (PI4KIIIα and PI4KIIIβ). PI4KIIIβ, together with Rab GTPases, plays a key role in regulation of membrane forward trafficking^3,4^, PI4KIIIα has been shown to regulate plasma membrane PI4P, while surprisingly not being a strong regulator of PIP_2_^5^. The role of PI4KIIIα on ion channel regulation has not being explored. Although PI4K inhibitors have recently been proposed as treatment of hepatitis C and malaria^6–9^, and powerful tools have been developed to study the role of PI4P in the plasma membrane, PI4P known role in the regulation of ion channels has been limited. To date a single study on PI4P regulation of ion channels revealed that PI4P is able to substitute for PI(4,5)P2 in the regulation of TRPV1 channels, while having no effect on TRPM8 channel^10^.

A decrease in the slow delayed rectifier potassium current (I_Ks_) due to mutations in the alpha subunit of the channel, KCNQ1, leads to prolongation of the QT interval on the ECG which underlies the development of inherited cardiac arrhythmias (LQT1). Phosphoinositols, including PI(4,5)P_2_ and PI4P, are negatively charged and known to interact with positively charged amino acids in KCNQ1^11,12^. A number of LQT1-associated mutations occur in phosphoinositide interaction domains^12,13^. Although the role of the phospholipid PI(4,5)P_2_ in stabilizing ion channel function, including KCNQ1/KCNE1, is well established, little is known about the role of phospholipids in channel membrane localization and the role of the PIP_2_ precursor PI4P or other phospholipids in channel current and localization. PI4P has been shown to directly bind the KCNQ1 subunit^12^. However, the roles of these interactions on cardiac electrophysiology are unknown.

Here we show that inhibition of PI4 kinases dramatically decrease KCNQ1/KCNE1 membrane localization. In addition, we show that decrease in membrane PI4P but not PI(4,5)P_2_ levels lead to channel internalization. Mutations associated with Long-QT syndrome in phospholipid interacting residues also show a dramatic dominant-negative decrease in channel membrane expression, while deletion of the distal C-terminus abolishes PI4K-inhibitor effects and rescues membrane expression of phospholipid-interacting Long QT mutant channels. Our results suggest that PI4P plays a role in stabilizing channel membrane localization. These results are the first to uncover a possible harmful effect of PI4K inhibition on cardiac electrophysiology and suggest the distal C-terminus of the channel is critical for PI4P regulation of the KCNQ1/KCNE1 channel.

## Results

### Inhibition of PI4 kinase leads to decrease cell surface expression of KCNQ1/KCNE1 channels

**Fig. 1:**
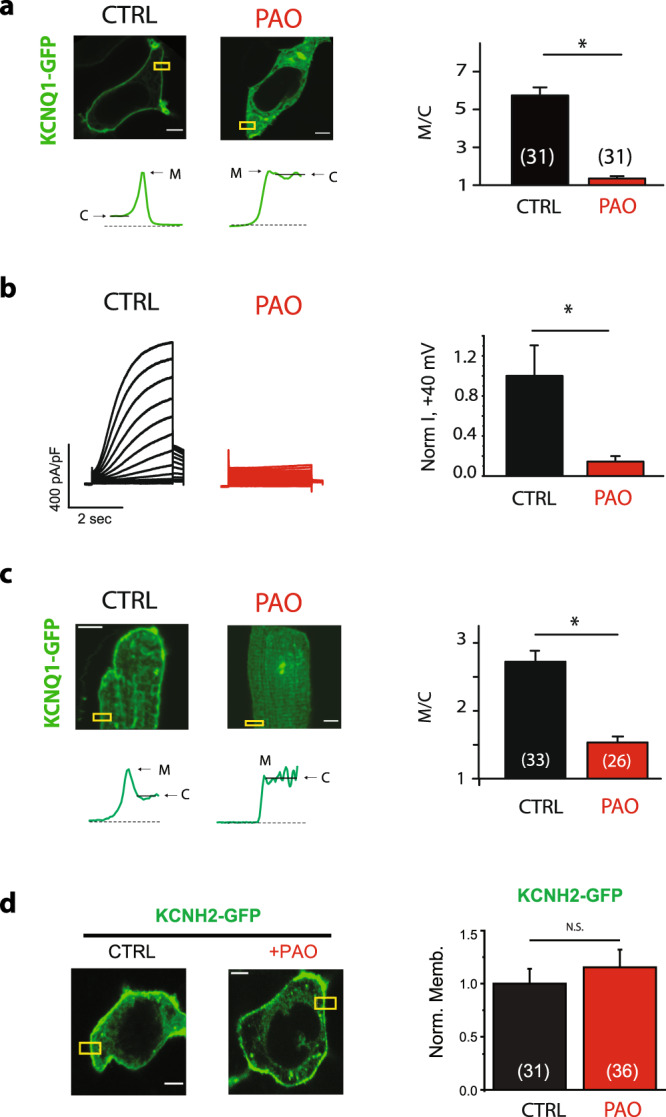
PI4K inhibition leads to decrease in channel membrane localization and current of KCNQ1/KCNE1. **a** Left: typical HEK293T cells expressing KCNQ1-GFP and KCNE1 treated with PAO (2 µM, 90 min) as indicated. Right: summary data of membrane to cytoplasmic fluorescence ratio (M/C) measured in experiments conducted as in the left panels. **b** Left: typical current traces recorded in HEK293T cells expressing untagged KCNQ1 and KNCE1 subunits treated with PAO (2 µM, 90 min) as indicated. Right: summary data (*n* = 6). **c** Left top: typical adult rat ventricular myocytes expressing KCNQ1-GFP and KCNE1 treated with PAO (2 µM, 90 min) as indicated. Left bottom: fluorescent profiles of KCNQ1-GFP expression in the boxed area. Right: summary data of sarcolemmal membrane to cytoplasmic fluorescence ratio measured in experiments conducted as in left panels. **d** Left: typical HEK293T cells expressing KCNH2-GFP treated with PAO (2 µM, 90 min) as indicated. Right: summary data. M = plasma (**a**) or sarcolemmal (**c**) membrane fluorescence; C = cytoplasmic fluorescence. Scale bars, 5 µM. *p < 0.05. number of cells indicated in parenthesis.

In order to study the role of PI4 kinase (PI4K) on channel localization we expressed C-terminal GFP-tagged KCNQ1 (KCNQ1-GFP) together with the untagged KCNE1 subunit in HEK293T cells. KCNQ1 localization was quantified as the ratio of membrane to cytosolic GFP-fluorescence in a cross-section of the cell using confocal images. Cells were treated with phenylarsine oxide (PAO), a PI4K inhibitor (Fig. 1a). Inhibitors of PI4K activity such as PAO have been shown to cause depletion of cellular PI4P, with only minor effects on the total amount of PI(4,5)P2^10,14^. PAO strongly inhibit channel membrane localization after 60 min treatment (Fig. 1a and Supplementary Fig. 1). In all, 30 min treatment had no significant effect on channel membrane localization (Supplementary Fig. 1). We also examined the effect of PAO on channel currents by expressing untagged KCNQ1 and KCNE1 subunits in HEK293T cells (Fig. 1b). We also examined the effect of PAO on channel current by expressing untagged KCNQ1 and KCNE1 subunits in HEK293T cells (Fig. 1b). PAO treatment strongly inhibited channel current (Fig. 1b and Supplementary Fig. 2). To investigate possible effects of PAO on channel gating we tested the functional effect of 60 min PAO treatment on channel current (Supplementary Fig. 2), we observed a significant inhibition of channel conductance, but no significant effect of PAO on channel voltage dependence of activation, consistent with the effect on channel membrane localization. Overall, our data indicate that PI4K inhibition decreased channel membrane localization and current.

We additionally tested the effect of Wortmannin (WMN) on channel membrane localization. WMN is an irreversible PI3 kinase inhibitor at low concentrations (IC_50_ = 1–10 nM)^15^ and an inhibitor of the PI4IIIK isoforms at 50–100-fold higher concentrations^16,17^. Low concentration of WMN (10 nM, 30 min) did not change channel localization. However, high WMN concentration (5 μM) promoted channel internalization (Supplementary Fig. 3). Overall, our data indicate that PI4K inhibition decreased channel membrane localization and current.

### Inhibition of PI4 kinase leads to decrease cell surface expression of KCNQ1/KCNE1 channels expressed in cardiomyocytes

We next investigated the effect of PAO in KCNQ1 localization in isolated adult ventricular rat myocytes. Cardiomyocytes were infected with human GFP-tagged KCNQ1 adenovirus, together with untagged human KCNE1 adenovirus. Cells were treated 24 h after infection for 2 h with PAO. KCNQ1 sarcoplasmic membrane localization was decreased by PAO treatment (Fig. 1c). Our results suggest that the effect of the PI4K inhibitor on KCNQ1 localization is conserved in cardiomyocytes.

### Inhibition of PI4 kinase does not affect cell surface expression of KCNH2 channels

We next investigated the effect of PAO on other ion channels. GFP-tagged channel subunits were expressed in HEK293T cells. Cells were treated 24 h after transfection with PAO. We first tested whether membrane localization for the other major cardiac repolarizing channel, KCNH2, was affected. KCNH2 membrane localization was not affected by the PAO treatment (Fig. 1d). We additionally tested other ion channels (Kir2.1, Kv2.1, and KCNQ4, Supplementary Fig. 4), and none of the channels tested were sensitive to PAO. Our results suggest that the effect of the PI4K inhibitor on KCNQ1 localization is specific to the KCNQ1 channel.

### Mutations in phospholipid interaction sites decrease cell surface expression of KCNQ1/KCNE1 channels

**Fig. 2:**
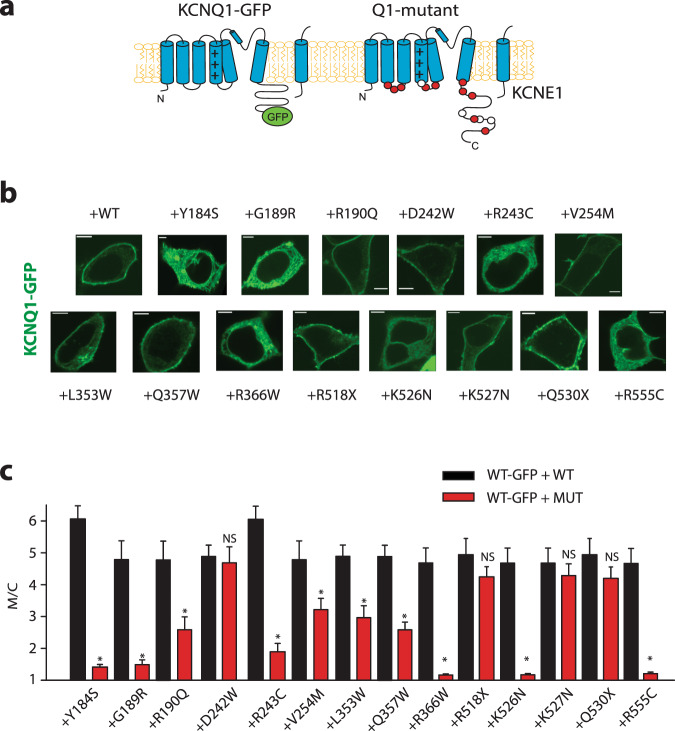
Expression of LQT1 mutant channel subunits causes a dominant negative decrease in channel membrane expression. **a** Scheme indicating the location of LQT1 mutant channels tested. Red circles indicate mutants that have a dominant negative effect on channel membrane localization, while white circles indicate mutations that do not affect channel membrane localization. **b** Representative confocal images of cells expressing KCNQ1-GFP, untagged mutant KCNQ1 and KNCE1 subunits, as indicated. **c** Summary data of membrane localization measured as ratio of membrane to cytoplasmic fluorescence (M/C) in cells expressing wild type and mutant channels as indicated. Scale bar, 5 µM, *p < 0.05, at least 20 cells for each condition.

Phospholipids have been shown to bind to cytoplasmic regions of the KCNQ1 subunit^18^. Here we investigated whether mutations in cytoplasmic domains affect channel plasma membrane expression. We focused on both common cytoplasmic mutations seen in patients^19,20^ and mutations on residues previously suggested to either interact or be in close proximity with phospholipids^21–23^. The majority of the studied mutations decreased membrane expression. In order to study the effect of cytoplasmic mutations on channel we tested the effect of 14 cytoplasmic mutants linked to Long QT syndrome type 1 on channel membrane expression (12 of the mutants tested were missense mutations and 2 deletion mutations, Fig. 2). We expressed the GFP-tagged KCNQ1 WT together with the untagged KCNQ1 mutant subunit and the untagged KCNE1 subunit (ratio 0.5:0.5:1.0). Expression of mutant KCNQ1 with KCNQ1 WT-GFP subunit decreased WT membrane localization in a dominant negative fashion for 10 of 12 of the missense mutants tested (Fig. 2).

To ascertain whether lack of the mutant expression in the plasma membrane was responsible for the lack of regulation of plasma membrane expression, we measured channel current for two mutants that show no decrease in plasma membrane expression when co-expressed with KCNQ1 WT (D242W and K527N). Previous work has shown that the D242W mutant channel shows current in the absence of KCNE1 expression^24^, thus, we measured channel current in the absence on KCNE1. Our results show that the mutant channel conductance is not significantly different from WT KCNQ1 current (Supplementary Fig. 5). For the K527N mutant we expressed the channel in the presence of KCNE1 and showed the channel conductance was also not significantly different from the WT KCNQ1/KCNE1 channel (Supplementary Fig. 5). Previous work had also showed K527N expressed current^25^. Our results are consistent with these mutant channels effecting gating and not channel membrane localization as previously published.

For mutants that show a dominant negative regulation of channel membrane expression, similar decrease in membrane expression were observed when the GFP-tagged mutant subunits were expressed alone in the absence of the KCNQ1 WT subunit (Supplementary Fig. 6). GFP-mutants also showed decrease in membrane expression in the presence of untagged WT channels (WT-GFP + WT: M/C:4.8 ± 0.3; R243C-GFP + WT: M/C = 1.25 ± 0.05; R555C-GFP + WT: M/C = 1.3 ± 0.1), suggesting both mutant and WT subunits membrane expression are inhibited. Note that most of the mutations that show decrease in membrane expression are located in known phospholipid interacting domains of the channel^18^.

### Deletion of the distal C-terminus of the channel inhibits PAO-mediated effects on cell surface expression of KCNQ1/KCNE1 channels

**Fig. 3:**
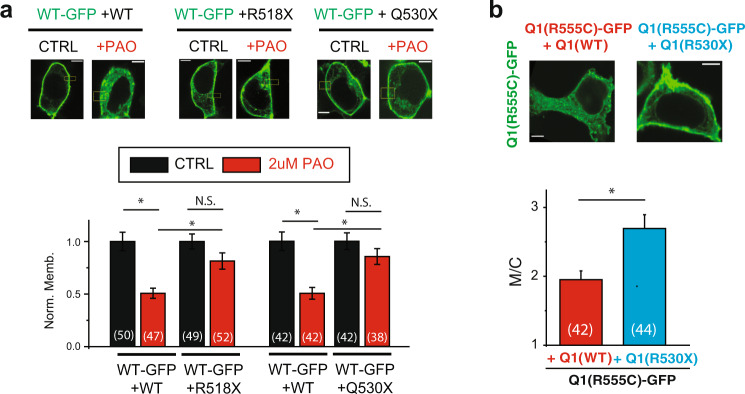
KCNQ1 deletion mutants abolishes PAO sensitivity when co-expressed with WT KCNQ1 and rescues membrane expression when co-expressed with R555C. **a** Top: typical HEK293T cells expressing KCNQ1-GFP, with and without untagged KCNQ1(Q530X) and KCNQ1(R518X) and KCNE1 treated with PAO (2 µM, 60–75 min), as indicated. Bottom: summary data of membrane to cytoplasmic fluorescence ratio (M/C) measured as in the top panels. **b** Top: typical HEK293T cells expressing KCNQ1(R555C)-GFP, together with either untagged KCNQ1(WT) or untagged KCNQ1(Q530X) and KCNE1, as indicated. Bottom: summary data of membrane to cytoplasmic fluorescence ratio (M/C) measured as in the top panels. Scale bars, 5 µM. *p < 0.05, (n) = number of cells.

In contrast to the dominant-negative effect of most C-terminal point mutations, channels containing KCNQ1 WT-GFP co-expressed with C-terminal truncation mutations (KCNQ1(Q530X) or KCNQ1(R518X)) showed robust surface expression (Fig. 2). Thus, we tested the effect of the expression of both KCNQ1(Q530X) and KCNQ1(R518X) subunit on KCNQ1(WT) channel regulation by PI4K inhibitors. In cells expressing either the C-terminal KCNQ1 truncation mutation (Q530X) or (R518X) together with the wild-type KCNQ1-GFP subunit, the channel regulation by PAO was blunted when compared to wild-type KCNQ1/KCNE1 membrane localization (Fig. 3a). In order to investigate whether deletion of the distal C-terminus could rescue the membrane expression of a phospholipid interacting mutant, we co-expressed KCNQ1(R555C)-GFP with either the KCNQ1(WT) or KCNQ1(Q530X) subunit (Fig. 3b). Channels expressing the deleted mutant subunit had significantly increased membrane localization when compared with the mutant channel co-expressed with the wild-type subunit. Taken together our data show deletion of the distal C-terminus abolishes PI4K inhibitor effects on channel membrane localization and can partially rescue the membrane expression of phospholipid interacting domain mutants, suggesting the distal C-terminus of KCNQ1 is critical for PI4P-mediated regulation of channel membrane localization.

### Decreased level of plasma membrane PI4P leads to decreased cell surface expression of KCNQ1

**Fig. 4:**
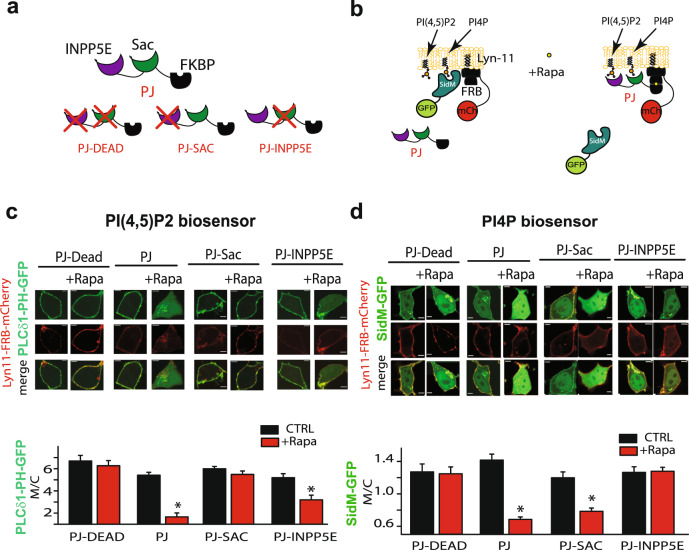
Rapamycin-induced membrane recruitment of PJ constructs specifically reduces PI(4,5)P_2_ and PI4P levels in the plasma membrane. **a** Schematic figure showing Pseudojanin (PJ), PJ-Sac, PJ-INPP5E, and PJ-dead constructs and the membrane targeted Lyn-11-FRB-mCherry construct. **b** Schematic figure showing Pseudojanin (PJ) and the membrane targeted Lyn-11-FRB-mCherry construct together with the PI4P biosensor SidM-GFP before and after rapamycin treatment. **c** Representative confocal images in HEK293T cells expressing PLCδ1-PH-GFP and the PJ constructs indicated (PJ, PJ-Sac, PJ-INPP5E or PJ-DEAD) before and after rapamycin treatment (1 μM, 30 min). **d** Summary data of PLCδ1-PH-GFP (top) and SidM-GFP (bottom) membrane to cytoplasmic fluorescence ratio (M/C) measured. Cells were treated as indicated. Scale bars, 5 μM. *p < 0.05. number of cells indicated in parenthesis.*p < 0.05.

To investigate the effect of changes in membrane phospholipid levels on channel membrane localization, we used a previously described rapamycin-induced heterodimerization system^10^. For these experiments, the FKBP-fused version of a bifunctional phosphatase pseudojanin (PJ) construct containing both 4- and 5-phosphatase domains was used. To specifically regulate either PI4P and PI(4,5)P2 levels we used constructs containing either the 4- phosphatase or 5-phosphatase domains. As controls we used a construct containing both 4- and 5-phosphatase domains (PJ) and a kinase dead construct (PJ-dead). FRB was targeted to the plasma membrane using the membrane targeting sequence of the Lyn protein (Fig. 4a). Effects of these manipulations on PI4P and PI(4,5)P2 levels were validated using biosensors based on specific lipid-binding domains: SidM as the PI4P sensor^26^ and PLCδ-PH as the PI(4,5)P_2_ sensor^27^. Treatment with rapamycin (30 min) induced PJ recruitment, resulting in a decrease in both PI4P and PI(4,5)P_2_ membrane levels. Constructs containing 4-phosphatase alone specifically changed PI4P levels and constructs containing 5-phosphatase alone specifically changed PI(4,5)P_2_ levels, while PJ-dead construct expression showed no effect on channel localization (Fig. 4b).

**Fig. 5:**
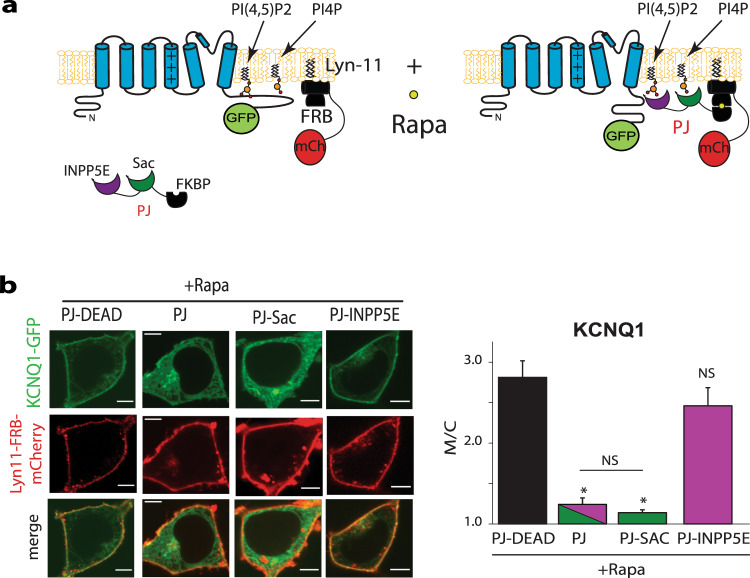
Decrease in PI4P but not PI(4,5)P_2_ levels leads to decrease in channel membrane localization. **a** Schematic figure showing Pseudojanin (PJ), PJ-Sac, PJ-INPP5E, and PJ-dead constructs and the membrane targeted Lyn-11-FRB-mCherry construct together with the channel before and after rapamycin treatment. **b** Left: representative HEK293T cells expressing KCNQ1-GFP, KCNE1, and the PJ constructs indicated after rapamycin treatment (1 μM, 30 min). Right: summary data of membrane to cytoplasmic fluorescence ratio (M/C) of KCNQ1-GFP measured in experiments as the left panel. Cells were treated as indicated. Scale bars, 5 µM. *p < 0.05, number of cells at least 27.

Next we treated cells co-expressing KCNQ1 and KCNE1 subunits together with the membrane FRB-Lyn11-target domain and each of the FRB-phosphatase constructs with rapamycin for 30 min to induce lipid phosphatase translocation and to investigate the effect of prolonged inhibition of phospholipids (Fig. 5b). Our results show that only after recruitment of the 4-phosphatase to the membrane, causing reduction on PI4P levels, but not when 5-phosphatase alone was recruited, and PI(4,5)P_2_ levels reduced, channel membrane localization was decreased (Fig. 5). Taken together, our data suggest that PI4K activity inhibits channel membrane localization via decreases in membrane levels of PI4P, leading to channel internalization.

### Increased level of plasma membrane PI4P leads to increased cell surface expression of mutant KCNQ1 channels

**Fig. 6:**
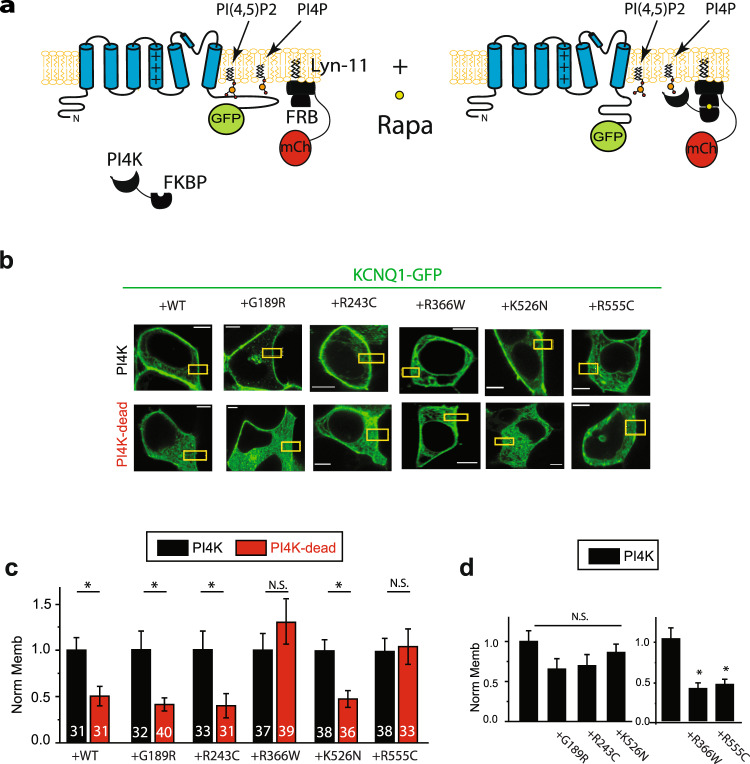
KCNQ1(R366Q) and KCNQ1(R555C) mutations abolish PI4K regulation of KCNQ1 plasma membrane localization. **a** Schematic figure showing PI4K-FKBP construct and the membrane targeted Lyn-11-FRB-mCherry construct. **b** Representative confocal images in HEK293T cells expressing KCNQ1-GFP and the PI4K constructs indicated (PI4K or PI4K-DEAD) after rapamycin treatment (1 μM, overnight). Number of cells indicated. **c** Summary data of KCNQ1-GFP membrane localization normalized to membrane localization observed in the presence of PI4K construct. **d** Summary data of KCNQ1-GFP membrane localization normalized membrane localization observed in the presence of the WT construct. At least 30 cells for each condition. Cells are treated as indicated. Scale bars, 5 μM. *p < 0.05.

To investigate the effect of changes in membrane phospholipid levels on mutant channel membrane localization, we used a FKBP-fused version of a construct containing either a constitutively active PI4-kinase or PI4-kinase dead constructs^28^. As the PJ constructs, FRB was targeted to the plasma membrane using the membrane targeting sequence of the Lyn protein (Fig. 6a). Effects of these manipulations on PI4P levels were validated using the biosensors SidM as the PI4P sensor^26^ Overnight treatment with rapamycin resulted in an increase of PI4P levels in cells expressing the PI4K constructs compared to PI4K-dead (Supplementary Fig. 7). Shorter rapamycin treatment did not affect SidM localization.

Next we treated cells co-expressing KCNQ1, KCNQ1 mutant and KCNE1 subunits together with the membrane FRB-Lyn11-target domain and each of the FRB-phosphatase constructs with rapamycin to investigate the effect of prolonged regulation of PI4P membrane levels on mutant membrane localization. Our results show the wild-type channel membrane localization was inhibited by the expression of the PI4P-dead construct when compared to the PI4P construct (Fig. 6b, c). Our results show that for the mutants tested located in the channel cytoplasmic loops (G189R and R243C) and for one of the C-term located mutants (K526N), the channel membrane localization was comparable to WT, suggesting increase PI4P levels rescued the mutant phenotype (Fig. 6d). Two of the mutants (R366W and R555C) abolished the sensitivity of the channel to PI4P, suggesting they are crucial to PI4P regulation. Taken together, our data suggest that decreased levels of PI4P underlie decrease in mutant membrane localization and R366 and R555 are essential to the channel regulation.

## Discussion

This work explored the role of specific phospholipids interactions on KCNQ1/KCNE1 membrane localization. We showed that the majority of the cytoplasmic missense Long QT type 1 associated mutations tested decreased channel membrane localization. We showed that PI4K inhibition and plasma membrane PI4P depletion, but not PI(4,5)P_2_ depletion, resulted in decrease in wild-type channel membrane localization. Our results indicate that the distal C-terminus is critical for this effect. We show that co-expression of a C-terminus truncation mutant subunit both abolished the PI4K-mediated inhibitory effect and co-expression of two C-terminus mutant subunits abolished PI4P regulation of membrane localization. Our results are the first to indicate a role of PI4P on ion channel membrane localization, and suggest that decrease in PI4P availability to the channel, either due to inhibition of PI4K or as consequence of a mutation, has profound consequences for channel membrane localization and current.

Membrane PI(4,5)P_2_ binding to cytoplasmic domains of the KCNQ1 subunit is critical for KCNQ1/KCNE1 channel function^18^. We and others have shown that mutations in KCNQ1 that affect binding to PI(4,5)P_2_ decrease channel function and increase sensitivity to agonist-mediated PI(4,5)P_2_ hydrolysis^11^. Residues in the cytoplasmic domains of KCNQ1 have been shown to bind to other phospholipids^12,22,29,30^, including PI4P^12^, although consequences of the interactions with PI4P have not been explored. Recently, acute depletion of PI4P from the plasma membrane was shown not to affect KCNQ1/KCNE1 function^31^. In contrast to our results, the same study showed that prolonged depletion of membrane PI4P did not affect membrane expression using an N-terminus KCNE1 tagged construct fused to the KCNQ1 subunits. We have recently shown KCNE1 is critical to KCNQ1 regulation of membrane localization mediated by PKCβII^32^. In our experience, tags to KCNE1 strongly affect its interaction with the KCNQ1 subunit and are likely responsible for the different results obtained. Consistently, we measured current regulation of the channel using a KCNQ1-KCNE1 tandem construct^33^ and observed a strong shift in voltage dependent of activation by PAO (V1/2 CTRL 44 ± 4, PAO 73 ± 10, *p* = 0.015), without observing a decrease in channel conductance (Gmax CTRL 1.00 ± 0.14, PAO 0.82 ± 0.23, *p* = 0.51), these results are in strong contrast to our data for the WT channel, suggesting regulation is affected by the link. Our results suggest an important novel role of PI4P on KCNQ1/KCNE1 channel membrane localization. PI4P may also be important for membrane localization of other PI4P-sensitive channels.

A number of mutations associated with Long QT syndrome are located in residues that affect channel interactions with phospholipids^18,25,29,30,34^, including PI4P^18^. For this study, we selected mutations in cytoplasmic regions to investigate their effect on channel membrane localization. These mutations have been shown to either change or be in close proximity to cytoplasmic charge shown to affect phospholipid interactions^21–23^. Here we show that cytoplasmic missense mutations tested show a consistent decrease in channel membrane expression (10/12 tested). Interestingly, the LQT mutations tested show a dominant negative, despite presumably not affecting all lipid-binding sites in the channel, suggesting intact interactions of all sites are necessary to stabilize channel membrane localization. ß-Blockers are the first line of therapy for patients with LQT1^35^. We have also shown that the current of LQT1-associated channels with mutations in phospholipid interacting residues is partially rescued by ß-adrenergic activation, suggesting that inhibition of ß-adrenergic signaling by ß-blockers may not be as effective in the treatment of these mutants^34,36^ Thus, alternative and/or additional treatment may be necessary for LQT1 patients with KCNQ1 mutations in phospholipid interacting sites. Here we show that deletion of the distal C-terminus of the channel abolish PAO regulation and co-expression with the KCNQ1(Q530X) subunit can rescue membrane localization of mutant channels with impaired trafficking. Consistently, patients with C-terminal truncation mutations at Q530X and R518X, show lower arrhythmic risk and modest QTc prolongation when compared to other Long QT patients^37,38^. Functional studies showed that the expression of KCNQ1(R518X) and KCNQ1(Q530X) mutants did not decrease the overall current when expressed with KCNQ1(WT)^39,40^. Our data show the distal C-terminus of the KCNQ1 channel is required for KCNQ1/KCNE1 channel regulation by PI4P. In particular, we show that two mutations in the C-terminus of the channel abolish channel regulation, suggesting they may be necessary for PI4P interactions. This is consistent with intact lipid interactions being necessary for channel membrane localization. The truncation mutations, KCNQ1(R518X) and KCNQ1(Q530X), may prevent the formation of a macromolecular complex and recruitment of proteins that are necessary for channel regulation^19,41–43^. We have previously shown that clinical phenotype of patients with these mutations are mild and basal current conductance are not significantly affected^39^. Although our results suggest that expression of these truncated mutants may rescue the mutant channel phenotype, readthrough of both R518X and Q530X mutants with premature termination codons were shown to rescue the R518X mutant but not Q530X mutant. These results are consistent with the larger deletion non-sense mediated decay also contributing to the patient phenotype^44^.

Although decrease in Golgi-PI4P levels has been acutely observed in response to hypertrophic stimulus^45^ and PI4KβIII activity and overall PI4P levels have been shown to increase in in intro and in vivo hypertrophy models^46^, the consequences of changes in PI4P levels to cardiac electrophysiology are currently unknown. Our studies indicate that further steps to investigate the effects of PI4K inhibitors to cardiac electrophysiology in vivo are important. Our data show that inhibition of PI4 kinase decreased KCNQ1/KCNE1 membrane expression and current. Decrease in KCNQ1/KCNE1 current is known to be associated with prolongation of action potential and cardiac arrhythmias. Although phospholipids have been shown to regulate ion channels in general, and cardiac channels in particular, the focus of most studies has been on gating effects of PI(4,5)P, while the role of its precursor, PI4P, has been overlooked. Here we uncovered that PI4P is a major regulator of KCNQ1/KCNE1 membrane trafficking. Although PI4K inhibitors have recently been proposed as treatment of hepatitis C and malaria^6–9^, the arrhythmogenic potential of these drugs has not been investigated. Our data indicate a potentially pro-arrythmic effect of PI4K inhibitors, prolonging cardiac repolarization via a reduction in repolarization reserve. This novel signaling pathway may have important consequences for arrhythmogenesis in inherited and acquired Long QT syndrome.

## Methods

### Constructs and chemicals

hKCNQ1, hKCNE1, hKCNQ1-GFP, and eGFP plasmids used were previously described^47,48^; Mutants KCNQ1(D242W), KCNQ1(L353W), KCNQ1(Q357W), KCNQ1(R366W), KCNQ1(K526N), and KCNQ1(K527N) were a generous gift from Dr. Bernard Attali **(**Department of Physiology and Pharmacology Sackler School of Medicine, Tel Aviv University, Israel)^49,50^. Mutants KCNQ1(Y184S), KCNQ1(G189R), KCNQ1(R190Q), KCNQ1(R243C), KCNQ1(V254M), KCNQ1(R555C)^47^ and KCNQ1(R518X), KCNQ1(Q530X)^39^ were cloned into pCDNA3.1 using site direct mutagenesis as previously described^11^. GFP-tagged constructs were fused with GFP at the C-terminus. Additional constructs used were Pseudojanin (PJ), a fusion protein of Sac and INPP5E phosphatase domains with FKBP (Addgene, #37999); PJ-Sac, a PJ based construct containing an inactivating mutation (Asp1263Ala) in the INPP5E domain (Addgene, #38000); PJ-INPP5E, a PJ based construct with an inactivating mutation (Cys779Ser) in the SAC1 domain (Addgene, #38001); PJ-DEAD, a PJ based construct containing an inactivating mutation (Asp1263Ala) in the INPP5E domain and an inactivating mutation (Cys779Ser) in the SAC1 domain (Addgene, #38002)^10,31^; PH-PLCδ1-GFP (Addgene, #51407); LYN11-FRB-mcherry (Addgene, #38004); GFP-P4M-SidM (Addgene, #51469), hKCNH2-EGFP (VectorBuilder); EGFP-Kir2.1 (Addgene, #107184); Kv7.4-EGFP (Addgene, #111453); Kv2.1-EGFP (Addgene, #111531); constitutively active PI4K (Addgene, #139311); dominant negative PI4K-dead (Addgene, #139312); KCNE1-KCNQ1 tandem^33^. The adenoviral constructs hKCNQ1-GFP, hKCNE1 (Vector Biolabs) were used to infect rat ventricular cardiomyocytes. Di-8-ANEPPS dye (Enzo, # 52204) was used to label the cells membrane. Phenylarsine oxide (PAO) (Santa Cruz Biotechnology, # sc-3521) and Wortmannin (Cayman Chemical Company, #19545-26-7) were used, all other chemicals were purchased from Sigma Aldrich.

### Cell culture and transient expression

HEK293T cells (ATCC: The Global Bioresource Center) were cultured in 35-mm petri dishes in DMEM (Corning Cellgro, 15-013-CV) supplemented with 10% FBS (Sigma-Aldrich) and 1%GlutaMax (Cellutron Life Technologies) under 5% CO_2_ at 37 °C. Cells were transiently transfected with the plasmid DNA of interest using FuGene HD following manufacturer’s instruction (Promega). Equal amount of plasmid DNA of KCNQ1 (or KCNQ1-GFP or its mutants) and KCNE1 were used. Confocal images and current recordings were conducted 48–56 h after transient expression of the channels.

HEK293T cells were transfected with GFP-tagged KCNQ1 (1.5 µg) and KCNE1(1.5 µg). 6 h after transfection, cells were plated on glass bottom dishes (MatTek Corporation). Forty-eight hours after transfection, cells were washed two times with PBS (Quality Biological) and incubated at 37 °C for treatment in extracellular recording solution (below). For all treatments, cells were incubated at 37 °C, 5% CO_2_. DMSO treated cells were used as control for PAO (2 μM) and WMN (0.01 μM, 5 μM) treatment. Rapamycin (1 μM) was applied for 30 min to induce PJ, PJ-SAC, PJ-INPP5E, or PJ-DEAD translocation to the plasma membrane. Confocal images and patch clamp experiments were performed at room temperature immediately after treatment.

### Adult cardiomyocyte isolation and cell culture

Animal care and procedures follow UCAR (University Committee on Animal Resources) guidelines at University of Rochester. Ventricular cardiomyocytes from 6–12-weeks-old SD (Sprague Dawley*)* female rat hearts were isolated as previously described^51–56^. Heart was removed and placed in ice-cold isolation solution (in mM: 133.5 NaCl, 4 KCl, 1.2 NaH_2_PO_4_, 10 HEPES, 1.2 MgSO_4_, 11 Glucose. pH value was adjusted to 7.4 with NaOH,). Heart was then cannulated via the aorta, and placed on the Langendorff perfusion system. All solutions were oxygenated (95% O_2_) during perfusion and kept at 37 °C. The hearts were initially perfused with calcium-containing (1 mM) isolation solution for 5 min followed by calcium-free isolation solution with added 0.1% BSA (Control Solution-BSA) for 5 min, followed by collagenase containing solution (0.09% w/v) for 20–30 min or until heart was soft to touch. Hearts were removed from the cannula and placed in a petri-dish with pre-warmed (37 °C) collagenase solution. Atria were removed, and the ventricular tissue was minced and cells isolated by gentle trituration with Pasteur pipettes of decreasing diameters. Cells were allowed to precipitate by gravity and calcium concentration was progressively increased in isolation solution (in µM: 50, 100, 200, 500, and 1000 CaCl_2_). Cells were plated in glass bottom dishes for 2 h, and incubated at 37 °C, 5% CO_2_. After 2 h, cells were infected with av-KCNQ1-GFP and av-KCNE1 (1 × 10^8^ p.f.u./ml each) overnight in 1 ml of culture media (M-199) supplemented with 1–2% penicillin/streptomycin. Cells were washed with fresh media daily.

### Electrophysiological data acquisition and analysis

For patch clamp experiments, HEK293T cells were transfected with 0.5 µg plasmid of untagged KCNQ1, 0.5 µg plasmid of KCNE1, and 0.1 µg EGFP (used as a transfection marker). Conventional whole-cell patch-clamp was conducted 48–56 h after transfection. Extracellular recording solution contained (in mM):145 NaCl, 5.4 KCl, 1.8 CaCl_2_, 1 MgCl_2_, 10 HEPES, 10 D-Glucose, pH adjusted to 7.4 with NaOH. The pipette solution contains (in mM): 130 K Aspartate, 11 EGTA, 1 MgCl_2_, 1 CaCl_2_, 10 HEPES, and 5 K_2_ATP, pH adjusted to 7.2 with KOH. Cells were held at −80 mV, a 3 s depolarizing step from −80 mV to +100 mV in 10 mV interval was applied, followed by a step to −20 mV. Data were acquired using Axopatch 200B (Axon Instruments) equipped with an A/D converter Digidata 1322 A (Axon Instruments) and recorded and stored in a PC computer using Clampex 10 software. Data were analyzed in Clampfit software. Peak tail currents were fit into a Boltzmann equation, G(v) = Gmax/[1 + exp(−(*V*−*V*_1/2_)/*k*)] for conductance Gmax, which reflects channel density on plasma membrane, and midpoint voltage *V*_1/2_, where channels are half-maximally activated. More details have been previously described ^47^.

### Statistics and reproducibility

All results are shown as mean ± standard error of the mean (SEM). For comparison of two groups a *t*-test was used. For comparison of more than two independent groups, one-way ANOVA, followed by Dunnet’s test was done. The significance level was set at *P* < 0.05.

### Reporting summary

Further information on research design is available in the Nature Research Reporting Summary linked to this article.

## Supplementary information

Supplementary Information

Description of Additional Supplementary Files

Supplementary Data 1

Reporting Summary

## Data Availability

The source data for the graphs and charts in the main figures is available as Supplementary Data 1. Any remaining information can be obtained from the corresponding author on reasonable request.
