# Supplementary material for: Membrane pools of phosphatidylinositol-4-phosphate regulate KCNQ1/KCNE1 membrane expression
Source: Commun Biol. 2021 Dec 14;4:1392. doi: 10.1038/s42003-021-02909-1 (PMC8671492; doi:10.1038/s42003-021-02909-1)
Supplement: Supplementary file 1 — Supplementary Information [file 42003_2021_2909_MOESM1_ESM.pdf]

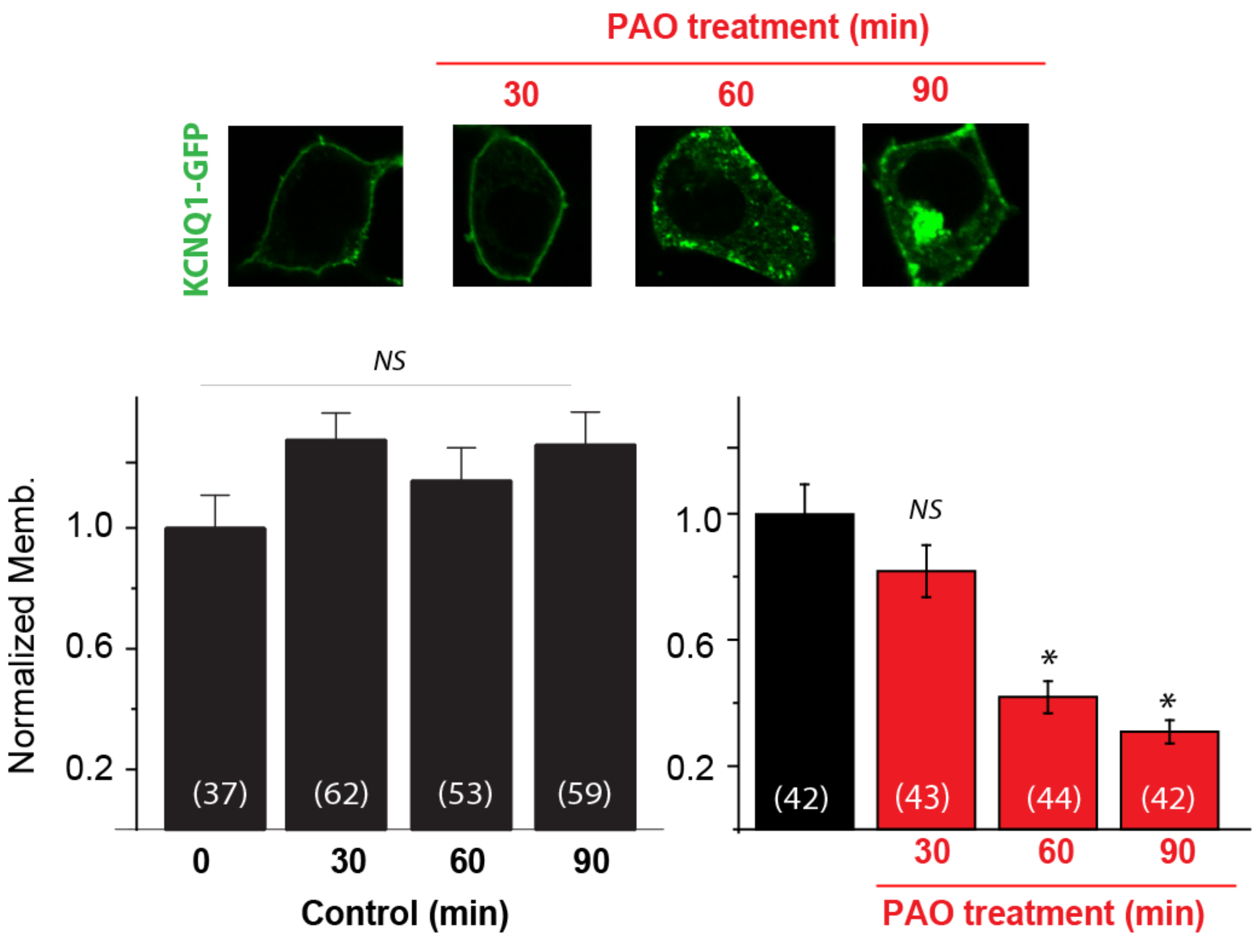


**Supplementary Figure 1: PI4K inhibition leads to decrease in channel membrane localization and current:** *Top*: Typical HEK293T cells expressing KCNQ1-GFP and KCNE1 treated with PAO (2uM) for different lengths of treatment, as indicated. Bottom: Summary data of membrane to cytoplasmic fluorescence ratio (M/C) measured in experiments as in the top panels. *, $p<0.05$, number of cells indicated in parenthesis.

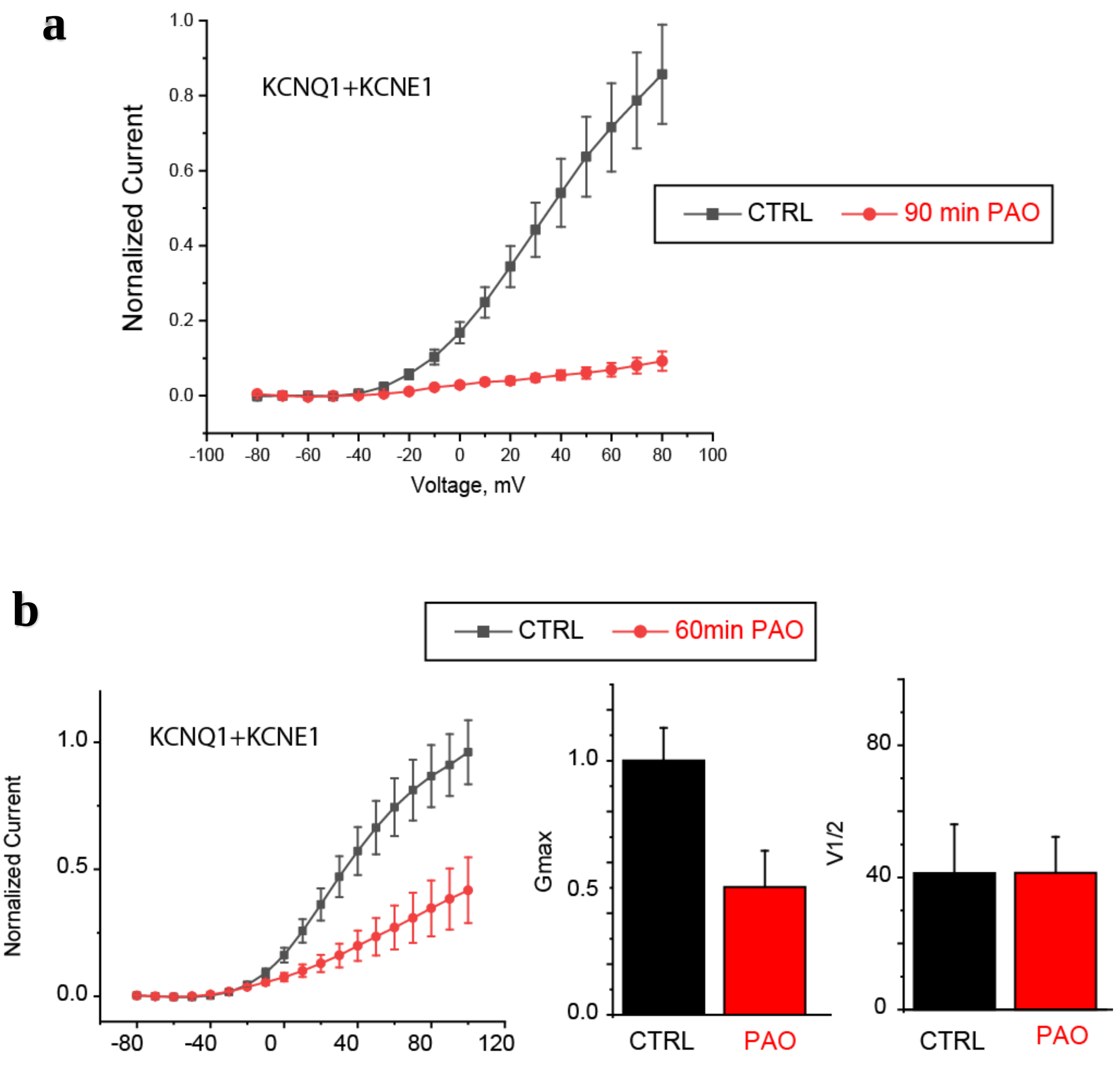


**Supplementary Figure 2: PI4K inhibition leads to decrease in channel current of KCNQ1/KCNE1. a)** *Left*: Average of IxV tail currents measured on HEK293T cells expressing untagged KCNQ1 and KCNE1 treated with PAO (2 µM, 90 min, n=6) as indicated. **b)** Average of IxV tail currents measured on HEK293T cells expressing untagged KCNQ1 and KCNE1 treated with PAO (2 µM, 60-75 min, n=12). *Right*: summary data of Gmax and V1/2 measured in individual cells (number of cells = 12).

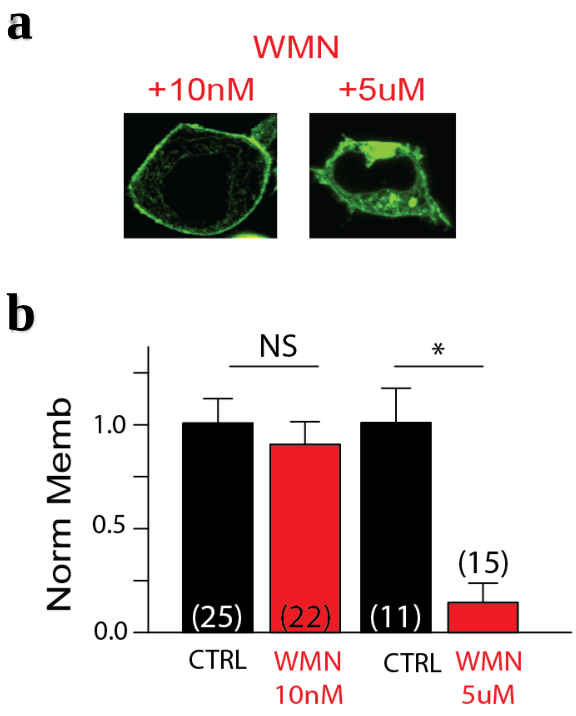


**Supplementary Figure 3: Wortmannin treatment leads to decrease in channel membrane localization at concentrations that block PI4K but not PI3K: a)** Typical HEK293T cells expressing KCNQ1-GFP and KCNE1 treated with wortmannin (30min) as indicated. **b)** Summary data of normalized membrane localization measured as in the top panels. *, $p<0.05$, number of cells indicated in parenthesis.

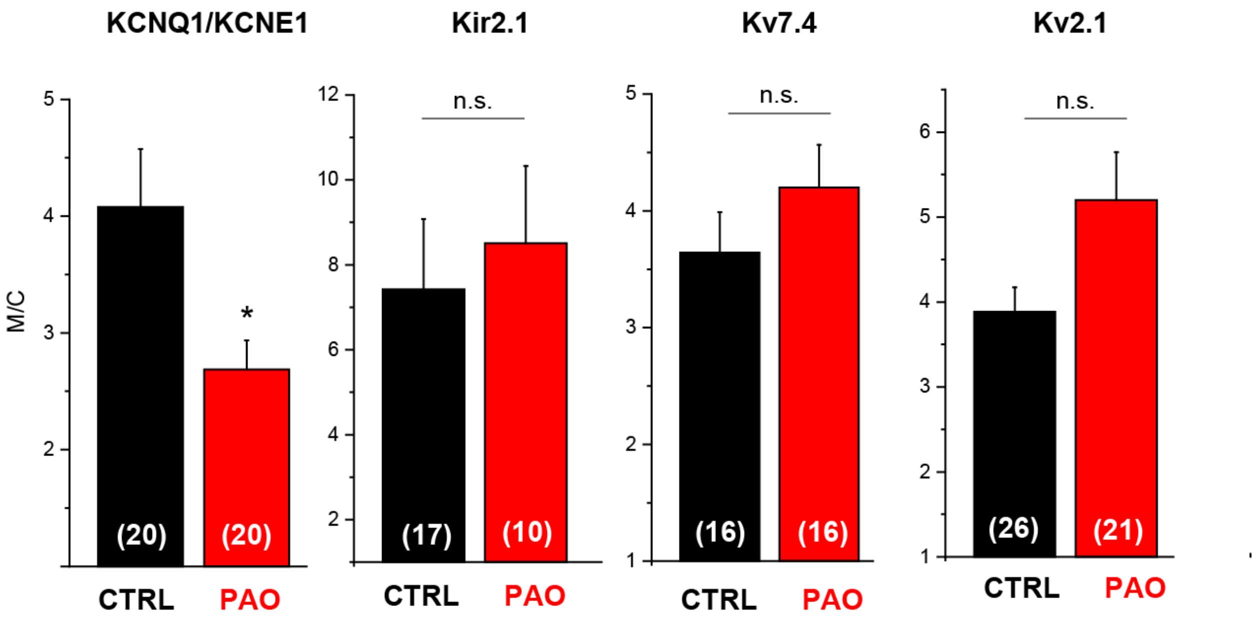


**Supplementary Figure 4: PI4K inhibition specifically decreased channel membrane localization for KCNQ1/KCNE1.** HEK293T cells expressing GFP-tagged channels and the treated with PAO (2 µM, 75 min) as indicated. Summary data of membrane to cytoplasmic fluorescence ratio (M/C). Number of cells indicated in parenthesis

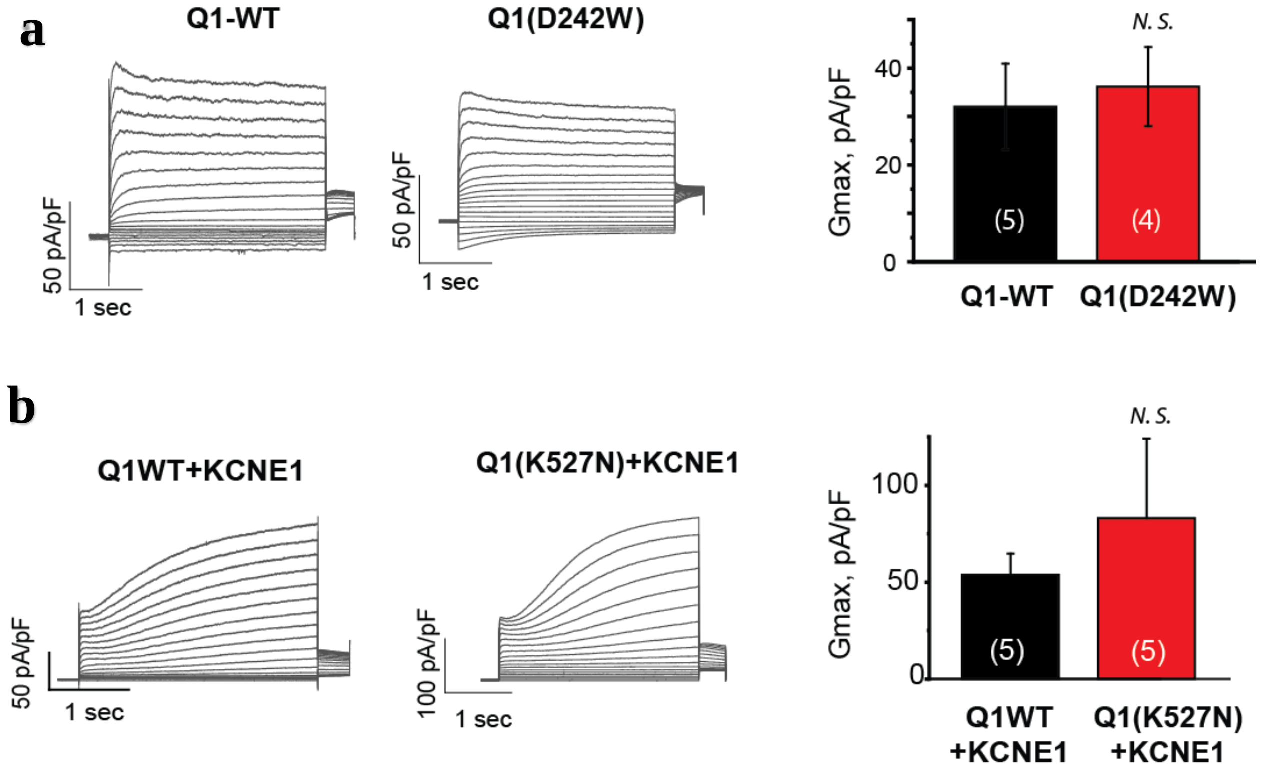


**Supplementary Figure 5. KCNQ1 mutants D242W and K527N both form functional channels.a)** *Left*: typical current recordings of a HEK293T cell expressing either KCNQ1WT or KCNQ1(D242W) in the absence of KCNE1 subunits; *Right*: Summary data of Gmax of the channels in the experiments conducted. **b)** *Left*: typical current recordings of HEK293T cells expressing KCNQ1WT or KCNQ1(K527N) and KCNE1; *Right*: summary data of Gmax measured in the experiments. Number of cells indicated in parenthesis

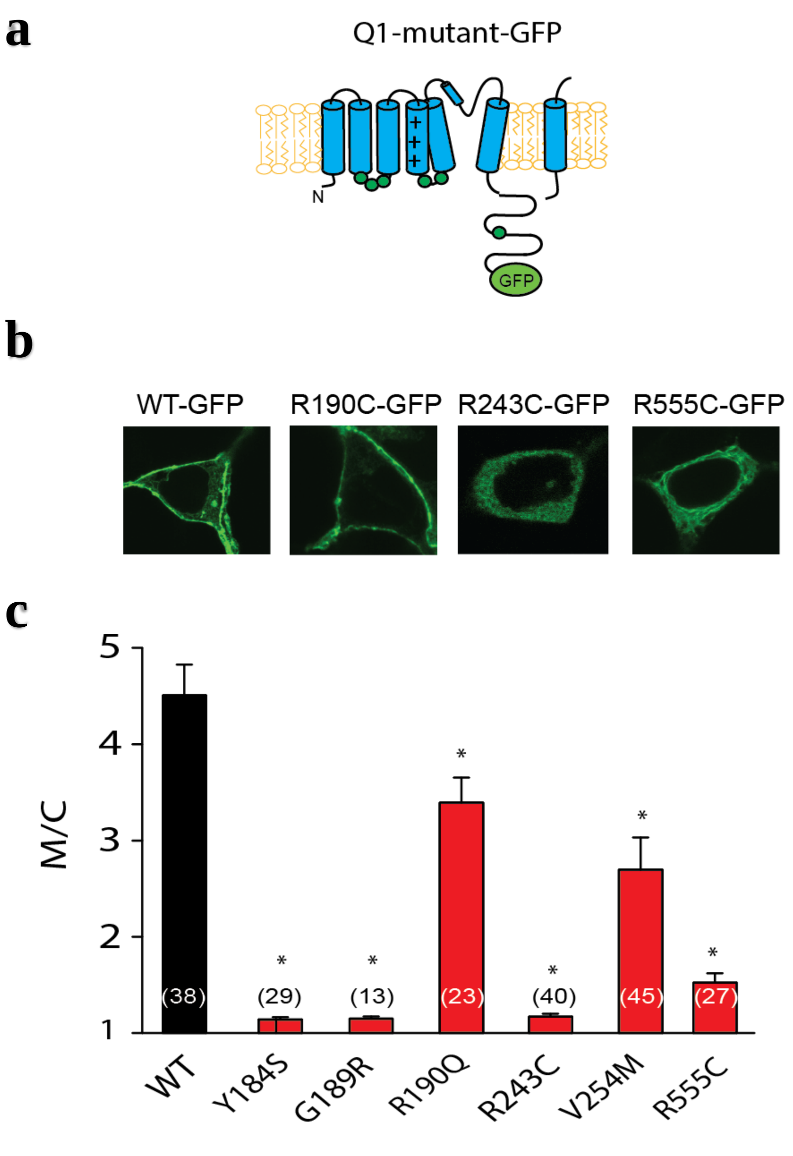


**Supplementary Figure 6: LQT1 mutant channel subunits show a decrease in channel membrane expression compared to WT KCNQ1 subunits. a)** Scheme indicating the location of LQT1 mutant channels tested. **b)** Representative confocal images of cells expressing GFP tagged mutant KCNQ1 and KCNE1 subunits, as indicated. **c)** Summary data of membrane localization measured as ratio of membrane to cytoplasmic fluorescence (M/C) in cells expressing wild type and mutant channels as indicated. *$p<0.05$, number of cells indicated in parenthesis.

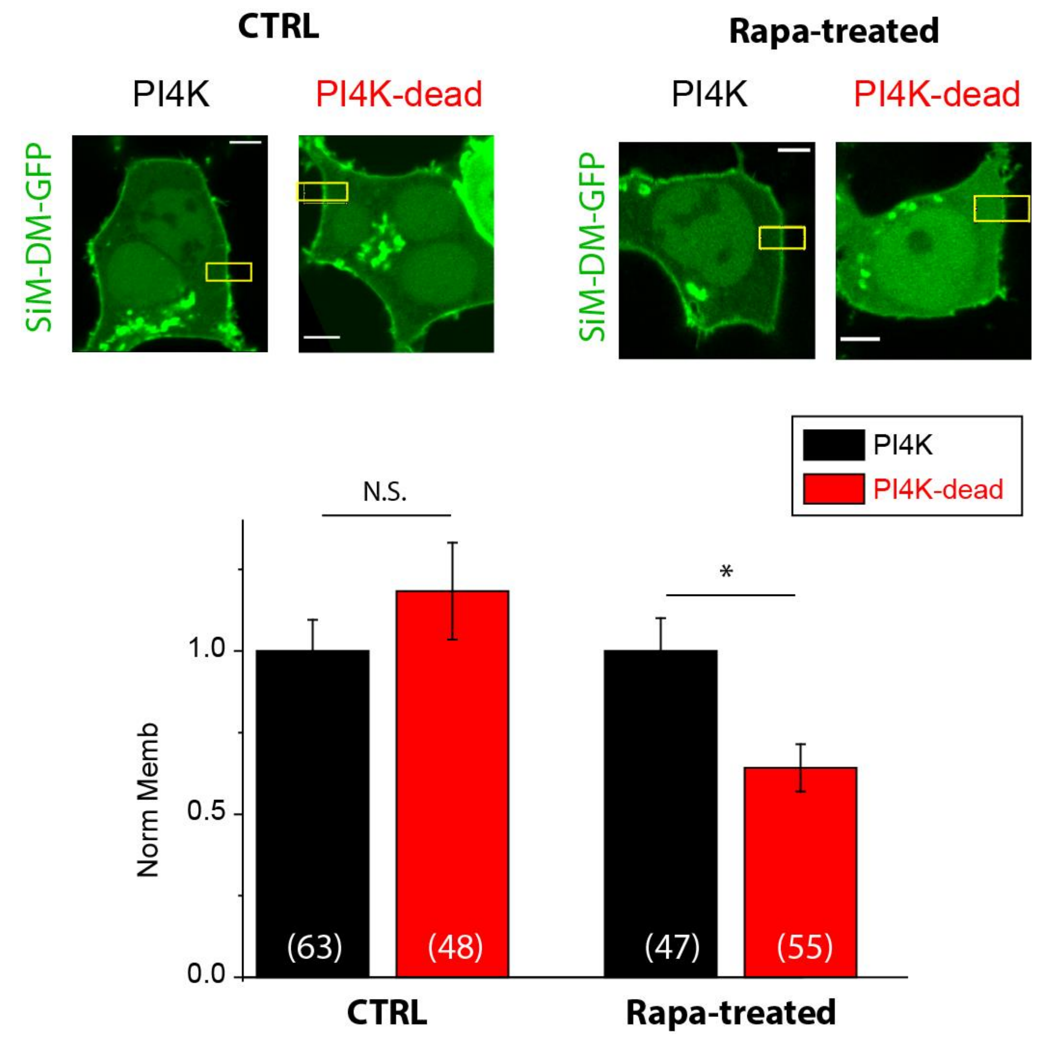


**Supplementary Figure 7. Rapamycin-induced membrane recruitment of PI4K construct increases PI4P levels in the plasma membrane when compared to PI4K-dead construct.** Top: Representative confocal images in HEK293T cells expressing SidM-GFP and the PI4K constructs indicated (PI4K or PI4K-DEAD) before and after rapamycin treatment (1 μM, overnight). Bottom: Summary data of SidM-GFP normalized membrane localization. Cells were treated as indicated. Scale bars, 5μM. *p<0.05, number of cells indicated in parenthesis.
