# Supplementary material for: Membrane pools of phosphatidylinositol-4-phosphate regulate KCNQ1/KCNE1 membrane expression
Source: Commun Biol. 2021 Dec 14;4:1392. doi: 10.1038/s42003-021-02909-1 (PMC8671492; doi:10.1038/s42003-021-02909-1)
Supplement: Supplementary file 2 — Description of Additional Supplementary Files [file 42003_2021_2909_MOESM2_ESM.pdf]

Description of Additional Supplementary Files

File name: Supplementary Data 1.
Description: Source data for figures.
